# Double Dart Technique of Instillation of Triamcinolone in Ganglion Over the Wrist

**DOI:** 10.4103/0974-2077.63286

**Published:** 2010

**Authors:** Dharmdas Paramhans, Dilip Nayak, Raj K Mathur, K Kushwah

**Affiliations:** *Department of Surgery, M.G.M. Medical College, Indore, Madhya Pradesh, India*; 1 *Department of Orthopedics, M.G.M. Medical College, Indore, Madhya Pradesh, India*

**Keywords:** Double dart technique, triamcinolone, wrist ganglion

## Abstract

**Background:**

Ganglia are the most common benign cystic swellings found over both the dorsal and volar aspects of the wrist. In spite of technical advancement, both operative and non-operative interventions achieve more or less similar results. Complete evacuation of gelatinous fluid followed with intra cystic instillation of triamcinolone has given encouraging results.

**Aims:**

To assess the efficacy and safety of drainage of cyst and instillation of triamcinolone in wrist ganglion.

**Materials and Methods:**

A prospective study was conducted on patients with simple ganglion cysts on the wrist. Total of 219 patients underwent this study. Out of this, 105 patients underwent the aspiration of the cyst fluid followed by intracystic instillation of triamcinolone, and 114 patients underwent surgical excision of wrist ganglia. Two years follow up was done for recurrence.

**Results:**

Most ganglia of wrist occurred in the extensor aspect. Complications noted among the surgically excised group were post operative pain and restricted mobility of wrist with a recurrence rate of 21.5%. Instillation of Triamcinolone into the ganglion yielded early resolution with a low recurrence of 8.4%.

**Conclusions:**

Intracystic instillation of triamcinolone after complete evacuation of cyst fluid is a simple and effective technique for treatment of ganglion.

## INTRODUCTION

Wrist ganglia are benign soft tissue tumors most commonly encountered on the dorsal aspect of the wrist and may communicate with the joint via a pedicle. Ganglia found on the volar aspect of the wrist attach via a pedicle to the radioscaphoid, scapholunate, scaphotrapezial joint, or the metacarpotrapezial joint.[1] They are cystic structures measuring 1-2 cm in diameter, feel much like a firm ball that is well tethered in place by its attachment to the underlying joint capsule or tendon sheath. Symptoms include pain in the wrist, which may radiate to the arm, and may be exacerbated by wrist movement, decreased range of motion, and reduced grip strength. Volar ganglia can also cause paresthesias from compression of the ulnar or median nerves or their branches.[2]

Surgery is the most common therapeutic option employed for treating ganglia and invovles excision of the entire ganglion complex, including cyst, pedicle, and a cuff in adjacent joint capsule. Recurrence may occur and is attributed to the remnants of the tortuous duct system located at the joint capsule due to inadequate dissection.

We describe a simple double dart method of instillation of triamcinolone as a safe and effective method for management of wrist ganglion.

## MATERIALS AND METHODS

A prospective study was conducted on patients attending outpatient department for simple ganglion cysts on the wrist from March 2001 to June 2009. Total of 219 patients were recruited in this study. Out of this, 105 patients underwent the aspiration of the cyst fluid followed by intracystic instillation of triamcinolone, and 114 patients underwent surgical excision of wrist ganglia. We included all the cases of wrist ganglia both on extensor as well as flexor aspect of wrist. More extensive ganglia like compound palmar ganglia were excluded. Cysts were examined for tenseness and cystic consistency by clinical examination.

**Figure 1:**
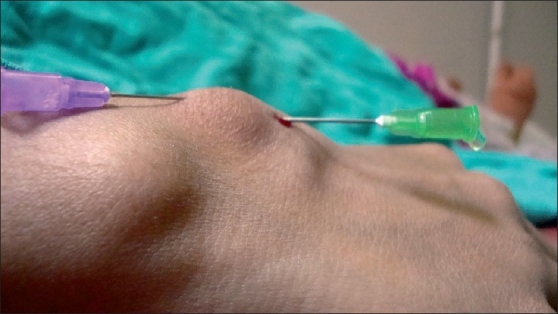
Cystic fluid being drained through the larger of the two needles inserted into the cyst can be visualized

**Figure 2:**
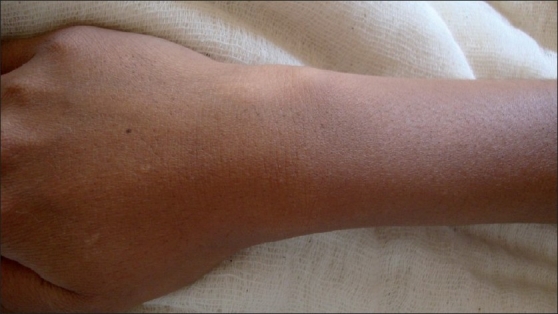
Site of wrist ganglion 1 year after treatment

A sterile wide bore needle of size 16 gauge and another of size 24 gauge hypodermic needles were inserted into the cyst wall facing each other [Figure 1]. The gelatinous content was allowed to escape through the wide bore needle by gentle compression of the cyst. After complete evacuation of cyst contents the wide bore needle was withdrawn and the puncture site was sealed with a swab. Triamcinolone acetonide 10%, 2 ml was injected into the cyst cavity via previously placed size 24 G. hypodermic needle, thus again refilling the cyst cavity to attain the similar size. Overstretching of cyst wall was avoided. This needle was now withdrawn and the puncture site was sealed with spirit swab. Patients were followed up every month for six months. Maximum follow up period was up to two years [Figure 2].

## RESULTS

Out of total 219 patients, 147 were females and 72 were males showing female preponderance. Age of patients ranged from 16 to 58 years with highest incidence noted in the age group between 20 to 30 years (87/219, 39.7%). Ganglia on extensor aspect were more common than on volar surface (181/219, 82.6%) in our study than volar ganglia. In triamcinolone group, there was mild pain locally for 48 hours which responded well to oral analgesics. None of them had any restricted wrist mobility, joint instability, or hypopigmentation of overlying skin. There was recurrence in 8 patients out of 105 (8.4%) within 6 months of the procedure, which responded to single repetition of local triamcinolone injection. There were no steroid induced side effects. Fourteen patients of surgical excision cohort had recurrence (21.5%). All these cases had post operative pain, lasting for four to five days and required analgesics. Wrist movements remained restricted for three to four weeks.

## DISCUSSION

Ganglia are localized, tense but often painless cystic swellings containing clear gelatinous fluid. They often communicate with and are always adjacent to a tendon sheath or the capsule of a joint. Their origin is uncertain, but they are probably caused by myxoid degeneration of fibrous tissue of capsule, ligaments and retinaculae. It consists of an outer fibrous coat and an inner synovial lining.[1] They occur singly but may be unilocular or multilocular. Ganglia is more common in women and often occurs between second and third decades (70%). Those occurring at distal interphalangeal (DIP) joints are associated with osteoarthritis and occur at older age. Spontaneous resolution is seen in 80% of children.

Ganglia are frequently located in the hand and wrist and less often in the ankle, foot and knee. In hand, 60-70% of all ganglia occur over scapholunate ligament. Nearly 20% of them present over volar aspect of wrist crease between flexor carpi radialis and abductor pollicis longus at the scaphotrapezoid joint.[2,3] There may be scapholunate diastasis that may persist even after excision.

Clinically, they present as slow growing localized swellings with mild aches. They are smooth, spherical and semi fluctuant to firm in consistency, with positive transillumination. They become more prominent on flexing the wrist. The extent of cyst and direction of pedicle can be well made out on compressing the cyst.[4]

Ganglia of the wrist may have a variable appearance on sonography like the presence of lobulations, well defined margins, internal echoes, and septations. Small ganglion cysts often appear hypoechoic without posterior acoustic enhancement, not appearing as simple cysts.[5]

Various management modalities are available. Simple technique of aspiration with a short beveled wide bore needle has been tried, with about 60% recurrence within 3 months.[6] Repeated aspirations gave 85% success rate. Many patients gave up the procedure during follow ups. Clay and Clement performed a radical excision of wrist ganglia by excising attachments up to scapholunate ligament. But this technique was associated with persistence of discomfort and recurrence with joint instability in one patient.[7] Regardless of whether the ganglion is removed via open surgery or arthroscopic excision, when a stalk is visualized, it should be completely removed, or the cyst should be removed at the origin to reduce the risk of recurrence. A recurrence rate of 13-40% has been observed following removal of entire cysts.[8]

A volar ganglion cyst is much more likely to recur than a dorsal ganglion cyst and care must be taken to protect the radial and ulnar neurovascular bundles because radial artery laceration is the most common vascular complication in volar carpal excision of ganglia. There was evidence of nerve damage to the superficial branch of the radial nerve and to the palmar cutaneous branch of the median nerve.[8] In open and arthroscopic procedures, wrist stiffness is the most common postoperative complication, along with infection, decreased motion, and ligament instability in open especially of the scapholunate ligament.[9–11] Arthroscopic resection of volar ganglion of wrist has been done with comparable results. In some, cyst wall was not removed and wide stalk has been left behind. The average operative time for such cases have been 55 minutes.[12] Even then, few recurrences have occurred. Rizzo reported stiffness in 25% of patients and required up to 8 weeks of occupational therapy to regain maximal function.

Local steroid infiltration in ganglion has shown satisfactory results in adults as well as in children.[14,15] Hyaluronidase also has been used to improve liquefaction in the cysts.[16,17] Steroids probably arrest the secretions from mesenchymal cells at the cyst wall. In our series evacuation of gelatinous fluid resulted in complete emptying of cyst cavity. Instilled triamcinolone, because of it's close contact with mucin secreting epithelium, leads to cessation of secretion of gelatinous fluid.

## CONCLUSION

We have evaluated a simple, noninvasive, safe and effective method of treatment for this common condition. This double dart procedure is scarless and therefore easily acceptable to patients. The absence of side effects such as wrist stiffness, joint instability or pain is an added advantage.
